# Treatment Modalities in Calcium Channel Blocker Overdose: A Systematic Review

**DOI:** 10.7759/cureus.42854

**Published:** 2023-08-02

**Authors:** Himanshi Baid, Nidhi Kaeley, Shiana Singh, Prakash Mahala, Hannah Chawang, Soumya Subhra Datta, Harsimran Manchanda, Takshak Shankar

**Affiliations:** 1 Department of Emergency Medicine, All India Institute of Medical Sciences, Rishikesh, Rishikesh, IND

**Keywords:** calcium channel antagonists, calcium channel blocker, overdose, toxicity, treatment modalities

## Abstract

Calcium channel blocker poisoning is one of the most common poisonings encountered which presents with life-threatening complications. However, there is no unified approach for treating these patients in the existing literature. This study aimed to assess the effects of different treatment modalities used in calcium channel blocker poisoning, as reported by previous studies. The primary outcomes studied were mortality and hemodynamic parameters after treatment. The secondary outcomes were the length of hospital stay, length of intensive care unit stay, duration of vasopressor use, functional outcomes, and serum calcium channel blocker concentrations.

A thorough literature search was performed through Ovid, PubMed, Cochrane Library, and Google Scholar from January 2014 to December 31, 2022, to identify all studies analyzing the effects of the treatment of calcium channel blocker poisoning on the desired outcomes. Two reviewers reviewed 607 published articles from January 2014 to December 2022 to identify studies analyzing the effects of the treatment of calcium channel blocker poisoning on desired outcomes.

In this review, 18 case reports, one case series, and one cohort study were included. Most patients were treated with an injection of calcium gluconate or calcium chloride. The use of calcium along with dopamine and norepinephrine was found to have lower mortality rates. A few patients were also treated with injection atropine for bradycardia. High-dose insulin therapy was used in 14 patients, of whom two did not survive. In the cohort study, 66 calcium channel blocker toxicity patients were included. These patients were treated with high-dose insulin therapy. A total of 11 patients with calcium channel blocker toxicity succumbed. Although it was found to be associated with improved hemodynamic parameters and lower mortality, side effects such as hypokalemia and hypoglycemia were noted. Intravenous lipid emulsion therapy (administered to eight patients), extracorporeal life support (used in three patients with refractory shock or cardiac arrest), injection glucagon, methylene blue, albumin infusion, and terlipressin were associated with a lower mortality rate as well as improvement in hemodynamic parameters. None of the case reports provided any information on end-organ damage on long-term follow-up.

## Introduction and background

Calcium channel blocker toxicity is reportedly the third fastest-growing substance exposure according to the American Association of Poison Control Centers, followed by beta-blocker overdose [1]. Verapamil is one of the most commonly reported calcium channel blocker overdoses [2]. As miscellaneous treatment regimens have been reported for calcium channel blocker poisons, formulating protocolized practice guidelines for calcium channel overdose is warranted.

A systematic review by St-Onge et al. [3] published in 2014 summarized the treatment for calcium channel blocker poisoning in the existing literature until December 31, 2013. Our systematic review aims to add to this systematic review by summarizing treatment modalities from January 2014 to December 2022. We have studied the reported effects of various treatment modalities used in calcium channel blocker toxicity on the desired outcomes of the patients managed for calcium channel blocked toxicity. The primary outcomes were mortality and hemodynamic parameters. The secondary outcomes were functional status, length of hospital stay, length of intensive care unit stay, duration of vasopressor use, and serum calcium channel blocker concentrations.

## Review

Methodology

Study Design

This systematic review included all case reports, case series, original articles, and abstracts from scientific and clinical meetings published from January 2014 to December 2022 on the treatment of calcium channel blocker toxicity. We defined a case report as an article that pertained to an individual case, or case series when two or more cases were reported.

Interventions

Studies with defined treatment strategies with an impact on primary or secondary outcomes were included in this systematic review.

Outcomes

The studies were required to study at least one of the primary and secondary outcomes. Primary outcomes included hospital mortality and hemodynamic parameters such as the improvement in blood pressure, stroke volume, heart rate, cardiac output, and peripheral vascular resistance. The secondary outcomes included the length of hospital stay, duration of intensive care unit stay, vasopressor use, and calcium channel blocker concentrations.

Search Strategy

**Figure 1:**
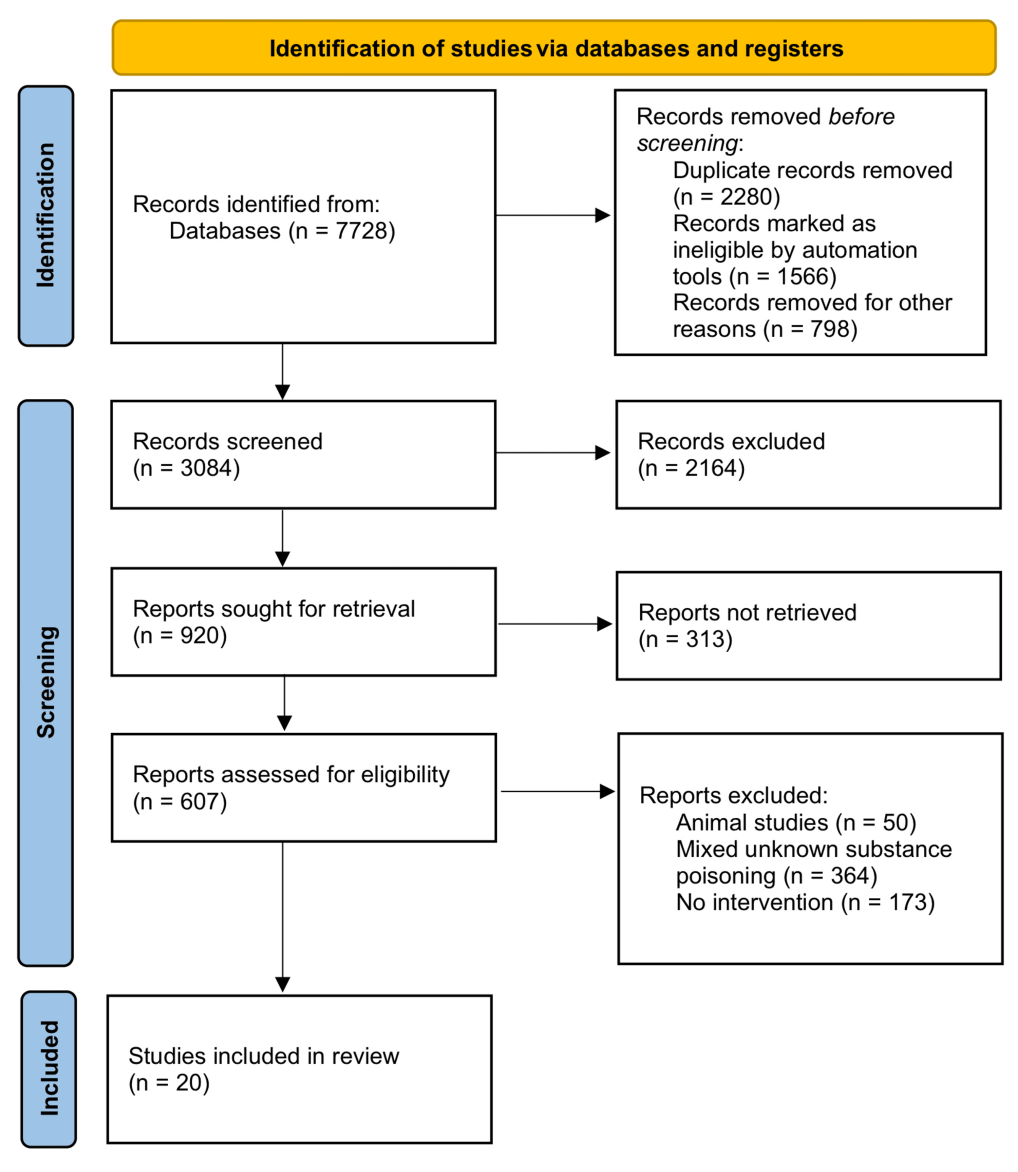
The Preferred Reporting Items for Systematic Reviews and Meta-Analyses flowchart.

A thorough search was made on Ovid, PubMed, Cochrane Library, Scopus, and Google Scholar from January 2014 to December 2022. Two researchers searched original articles, case series, and case reports using the keywords [calcium channel blockers OR calcium channel antagonist OR calcium channel blocking agent OR (amlodipine or bencyclane or bepridil or cinnarizine or felodipine or fendiline or flunarizine or gallopamil or isradipine or lidoflazine or mibefradil or nicardipine or nifedipine or nimodipine or nisoldipine or nitrendipine or prenylamine or verapamil or diltiazem)] AND [overdose OR medication errors OR poisoning OR intoxication OR toxicity OR adverse effect]. Animal studies were excluded from this review. Disagreements on the final articles included were resolved by a third reviewer. For data collection, a single flow sheet was used which included detailed study characteristics such as year of publication; study type; authors; demographic history of the subjects; history of comorbidities; treatment given; type, dose, and route of calcium channel blocker exposure; and primary and secondary outcomes. Quality assessment was done using Strengthening the Reporting of Observational Studies in Epidemiology for the original article. As the studies, interventions, and outcomes were heterogeneous, a planned meta-analysis could not be performed. After a thorough search, a total of 607 articles were identified, of which 20 articles were included. One was an original article, one was a case series, and 18 were case reports (Figure 1).

Results

Table 1 shows the results of individual studies.

**Table 1:** Individual studies and their outcomes. APTT = activated partial thromboplastin time; CCB = calcium channel blocker; COPD = chronic obstructive pulmonary disease; CRRT = continuous renal replacement therapy; CVVHD = continuous venovenous hemodialysis; CVVHDF = continuous venovenous hemodiafiltration; ECMO = extracorporeal membrane oxygenation; GCS = Glasgow Coma Scale; GI = gastrointestinal; HIET = high-dose insulin euglycemic therapy; IABP = intra-arterial blood pressure; ICU = intensive care unit; ILE = intravenous lipid emulsion; IU = international unit; IV = intravenous; KCl = potassium chloride; MAP = mean arterial pressure; NAC = N-acetylcysteine; PCM = paracetamol; RR = respiratory rate; SBP = systolic blood pressure; SPAD = single-pass albumin dialysis; VAD = ventricular assist device

Authors	Title	Study design	Participants	Intervention	Outcomes
Nasa et al. [4]	Continuous venovenous hemofiltration along with charcoal hemoperfusion for the management of life-threatening lercanidipine and amlodipine overdose	Case report	A 69-year-old man with hypertension, diabetes, and coronary artery disease presented with a 300 mg lercanidipine overdose and 50 mg amlodipine overdose	The patient was started on fluid boluses and 2 g of intravenous calcium gluconate. Gastric lavage was done with 70 g charcoal, followed by polyethylene glycol. IV dopamine was started and a central venous line was secured. Fluid resuscitation was continued along with vasopressors and calcium gluconate infusion at 0.5 g/hour. He was also given 40 units of insulin along with 50% dextrose and another 40 units of insulin was repeated after 30 minutes. The patient was started on charcoal hemoperfusion without ultrafiltration, with a 1 U/mL heparinized extracorporeal circuit for over four hours, followed by CVVHD with charcoal hemoperfusion	The patient’s condition started improving, with an increase in MAP around four hours after starting CVVHDF, with the urine output improving to 50 mL/hour after six hours. Another session of charcoal hemoperfusion was given after four hours. The vasopressors were progressively weaned off in the next 30 hours and CVVHDF was stopped in view of adequate urine output with resolution of metabolic acidosis. The glucagon infusion was continued at 5 mg/hour for 48 hours and then stopped. The IABP was removed 62 hours after insertion and the patient was extubated on day 4, shifted to the ward on day 6, and discharged the next day
Koliastasis et al. [5]	Refractory shock from amlodipine overdose overcomed with hyperinsulinemia	Case report	A 72-year-old female with no comorbidities. Amlodipine (200 mg) overdose with no co-ingestion	IV normal saline and 60 g of activated charcoal along with mechanical ventilation, followed by noradrenaline infusion (up-titrated at 1 µg/kg/minute) with 10% calcium chloride infusion (15 mg/hour), followed by high-dose insulin (45 IU/hour) along with 10% dextrose, up-titrated to 60 IU/hour. Calcium chloride infusion was stopped after 3 days. Insulin infusion and noradrenaline were down-titrated with hemodynamic monitoring and terminated after 5 days	After 30 minutes, SBP improved by 30 mmHg. She was weaned from ventilatory support after 8 days and discharged after 14 days. Follow-up at 1 month showed no cardiovascular comorbidity
Ramanathan et al. [6]	Extracorporeal therapy for amlodipine poisoning	Case report	A 25-year-old male with no comorbidities. Amlodipine 550 mg overdose with co-ingestion of paracetamol 13 g	The patient was intubated and mechanically ventilated, and a 2 L IV fluid bolus was given. Initiated on noradrenaline, adrenaline, and vasopressin to maintain a MAP of more than or equal to 60 mmHg. Activated charcoal was given nasogastrically. He was then given 5 mg IV glucagon and 40 mmol of calcium gluconate. He was then initiated on insulin (1 IU/kg/hour) and 120 mg IV methylene blue. He was then initiated on veno-arterial ECMO (flow of 4.5 L/minute, sweep gas flows of 4 L/minute, fraction of oxygen delivered was 0.8). Heparin anticoagulation was started to maintain an APTT target of 45–60 seconds. Therapeutic plasma exchange was also initiated. The inotropic support was weaned and withheld within 12 hours of plasma exchange and 24 hours of ECMO initiation. ECMO was weaned off and withheld on the 3rd day. He developed a fever and was started on antibiotics on day 1 of decannulation from ECMO. NAC was not started as PCM levels were below the treatment line of the *Rumack*–*Matthew nomogram*	The patient became hemodynamically stable after 12 hours of plasma exchange and 24 hours of ECMO initiation. He was weaned off ECMO on the 3rd day and extubated on the 4th day. He was shifted to nasal cannula oxygenation 12 hours post-extubation. He was later shifted to the ward and discharged after 27 days of hospital stay.
Van Veggel et al. [7]	A critical note on treatment of a severe diltiazem intoxication: high-dose calcium and glucagon infusions	Case report	A 53-year-old female with epilepsy, diabetes mellitus, coronary microvascular disease, COPD, alcohol abuse, and social problems. Diltiazem retard (4,800 mg)	The patient was given three doses of atropine, and multiple fluid challenges and isoprenaline infusions were started. This was followed by the initiation of noradrenaline (0.54 µg/kg/minute), dobutamine (8–13 µg/kg/minute), and adrenaline (0.04–0.06 µg/kg/minute) infusion along with intubation and mechanical ventilation. Calcium gluconate (1,000 mg) and insulin infusion (up to 0.15 IU/kg/hour) was then given for hyperkalemia. GI decontamination was done with magnesium sulfate and activated charcoal (even after 3.5 hours, as it was a retard formulation). A 3,000 mg bolus of calcium gluconate was given, followed by infusion at a rate of 6,000 mg/hour which was reduced to 3,000 mg/hour and stopped when the calcium level reached 5.05 mmol/L. Glucagon 5 mg followed by 1 mg infusion was also started. All cardiovascular support was stopped after 10 hours of initiation and glucagon after 16 hours of initiation. She was given calcitonin on the 2nd day in the ICU for pancreatitis. IV fluids and analgesics were continued	The patient’s hemodynamic status improved gradually over 10 hours when her cardiovascular support was withheld. She developed acute necrotizing pancreatitis on day 2 of the ICU stay (probably iatrogenic hypercalcemia or due to chronic alcohol use) which was also successfully treated
Chudow and Ferguson [8]	A case of severe, refractory hypotension after amlodipine overdose	Case report	A 53-year-old male with hypertension and depression with amlodipine 800 mg overdose	The patient was administered a bolus of 1 L normal saline, glucagon 1 g IV, and 2 g calcium gluconate IV. 220 units (2 units/kg) of regular insulin IV and then started on a continuous IV infusion at a rate of 220 units/hour (2 units/kg/hour). Noradrenaline infusion was started and the patient was intubated after 20 minutes due to altered mental state. Dopamine was added approximately 90 minutes later. Over the next 2 hours, the norepinephrine infusion was increased to the institution’s maximum rate of 0.3 µg/kg/minute. During this timeframe, he also received two 20% lipid boluses of 150 and 250 mL, respectively. At this point, both epinephrine and vasopressin were started. Over the next 2 hours, he received an additional 2 g of calcium chloride and 250 mL of 20% lipids. Phenylephrine was then started and was titrated to the institution’s maximum rate of 5 µg/kg/minute within 20 minutes. Methylene blue was started in an attempt to achieve hemodynamic stability. He received a 100 mg dose of methylene blue that was repeated approximately 1 hour later. He also received 2 hours of plasma exchange therapy after which he was placed on CRRT. Over the next 2 hours, he received an additional 250 mL of 20% lipids and 2 g of calcium chloride. In total, the patient received 14 g of calcium	The patient remained hypotensive during the insulin infusion, with blood pressures ranging from 81/41 to 57/40 mmHg. The patient remained hypotensive (SBP in the low 80s and MAP in the 50s) while on maximum rates of both norepinephrine and dopamine. The SBP increased to the low 90s and the MAP improved to the low 60s after the initiation of methylene blue. Approximately 2 hours after the second methylene blue dose, the beneficial effects of this agent began to wear off, and the patient’s SBP and MAP decreased to the low 80s and the 50s, respectively. Due to persistent hypotension, it was decided to place the patient on arterial–venous ECMO. As the patient was being transferred to the operating room table and prepped for the procedure, he became bradycardic and then asystolic and expired
Agarwal et al. [9]	The potential detrimental effects of calcium channel blockers’ overdose and current available management	Case report	A 51-year-old male with a past history of hypertension presented with a 500 mg amlodipine overdose and a self-inflicted abdominal knife wound in an apparent suicide attempt	The patient was intubated in the field and put on mechanical ventilation in the hospital. His initial treatment included sodium bicarbonate, dobutamine, dopamine, epinephrine, norepinephrine, vasopressin, lipid emulsion, insulin with dextrose infusion, calcium chloride, hydrocortisone, and electrolyte correction. A ventricular assist device (VAD)—Impella CP 4.0 (delivering 4 L of cardiac output (CO) per minute), was placed	The patient’s vitals and labs continued to deteriorate despite the use of multiple pressors and critical care medications. Imaging demonstrated no significant intra-abdominal trauma and hemoglobin levels remaining stable, both ruled out the self-inflicted knife wound as an explanation for hemodynamic instability. The patient’s clinical condition continued to worsen despite VAD placement. The further hospital course was complicated with critical limb ischemia and progression of multiorgan system dysfunction. Eventually, the patient’s family opted for Do Not Resuscitate orders and, unfortunately, the patient expired
Alkhatatneh and Elsayed [10]	High dose amlodipine poisoning treated with high insulin euglycemic therapy	Case report	A 72-year-old female with macular degeneration, cataract, depression, and hypertension. Amlodipine 500 mg poisoning	Initiation of HIET, calcium gluconate infusion, and vasopressor support within the first 4 hours after presentation, followed by a continuous infusion of insulin with D10 and calcium gluconate for 3 days	The patient improved within 4 hours of therapy initiation. She became normotensive on day 6 requiring no support
Cole et al. [11]	High dose insulin for beta blocker and calcium channel blocker poisoning	Original article (STROBE- 15/22)	199 patients with CCB toxicity (66), beta-blocker toxicity (88), and both CCB and beta blockers (45)	High-dose insulin therapy (insulin infusion >0.5 U/kg/hour or 25 U/hour)	Hypokalemia occurred in 29% of patients. Hypoglycemia occurred in 31% of patients; 50% (29/50) experienced hypoglycemia when dextrose infusion concentration was ≤10%, and 30% (31/105) experienced hypoglycemia when dextrose infusion concentration was ≥20%. A total of 41 patients experienced a cardiac arrest at some point in their care
Cumpston et al. [12]	Adjunctive use of low-dose intralipid associated with hemodynamic improvement in combined amlodipine and labetalol overdose refractory to standard therapy	Case report	A 55-year-old man with unknown quantities of amlodipine and labetalol overdose	The patient underwent tracheal intubation and resuscitation which included intravenous therapy with 2 L of normal saline, 9 mg of glucagon boluses and infusion at 7 mg/hour, 4 g calcium gluconate, 2 g calcium chloride, and norepinephrine titrated to 40 mg/minute. Hyperinsulinemia–euglycemia therapy was titrated to 250 units/hour with dextrose infusion. 20% intravenous ILE was started at 0.25 ml/kg/minute and continued for 3 hours. After 2 hours of completion, another dose of ILE at 0.17 mg/kg/minute was initiated and 5 hours after 2nd dose completion, a third dose of ILE at 0.04 mg/kg/minute was administered for 30 hours	Within minutes of starting ILE, the patient’s blood pressure increased to 92/52 mmHg. The patient recovered after three doses of ILE
Weinberg et al. [13]	Venoarterial extracorporeal membrane oxygenation for the management of massive amlodipine poisoning	Case report	A 50-year-old man with a history of depression and alcohol abuse presented with 500 mg of amlodipine, 1,000 mg of lisinopril, and 625 mg of hydrochlorothiazide overdose	The patient was intubated within 1 hour of arrival and started on dopamine, norepinephrine, normal saline, calcium gluconate, and glucagon, followed by initiation of phenylephrine, epinephrine, and vasopressin. As no improvement was seen, methylene blue and intravenous fat emulsion were added and he was started on veno-arterial ECMO and was decannulated from ECMO after 8 days	The patient did not improve despite maximum vasopressor therapy. The patient started improving after the addition of methylene blue and intravenous emulsion and after starting ECMO. His hemodynamic status improved, and his left ventricular function improved to 45% during his hospitalization. The patient was weaned off mechanical ventilation and supplemental oxygen on day 17. He was discharged from the hospital with an intact mental status on hospital day 56 and returned to functional independence
Raj et al. [14]	Amlodipine (150 mg) poisoning: a case study	Case report	An 18-year-old, female with no comorbidities. Amlodipine (150 mg) overdose	2,000 mL IV fluid with normal saline, followed by dobutamine (2.5 µg/kg/minute) and Noradrenaline (5 µg/kg/minute) up-titrated to target MAP >70 and urine output >0.5 mL/kg/hour. 270 mg IV bolus of calcium gluconate given over 15 minutes, followed by 90 mg/hour continued for 4 days, the dose was then reduced to 45 mg/hour up to day 5 (total dose 6.68 g). A glucagon injection of 2.5 mg IV bolus was given, followed by 5 mg/hour for 2 hours (could not be continued due to non-availability, total dose 12.5 mg). On day 2, she was started on high-dose insulin (50 IU bolus followed by 25 IU/hour infusion), along with 25 % dextrose at 100 mL/hour. It was up-titrated to 50 IU/hour on the 3rd day. This was reduced to 25 IU/hour on day 4 and stopped on day 5. Vasopressors were also tapered from day 4 and stopped on day 5	The patient improved after initial measures for 24 hours maintaining MAP (70 mmHg), RR of 24, and adequate urine output. She deteriorated on day 2 and hypotension persisted until the 3rd day. The patient stabilized after increasing the insulin infusion to 50 IU/hour. The patient showed good hemodynamic response over the next two days. She was shifted from the ICU on day 6 and discharged from the ward on the 8th day. No follow-up details were available
Fadhlillah and Patil [15]	Pharmacological and mechanical management of calcium channel blocker toxicity	Case report	A 64-year-old male with no comorbidities. Nimodipine 840 mg with co-ingestion of metformin 42 mg, risperidone 88 mg, pravastatin 840 mg, and ranitidine 9 g	Peripheral ephedrine, 4 doses of 500 µg atropine, and 2 boluses of 1,000 mL warm saline were given initially. Aliquots of adrenaline (100 µg) were then given, followed by an infusion of epinephrine (0.10 µg/kg/minute) and dobutamine (3.13 µg/kg/minute). He also received 30 mL of 10% calcium gluconate, and 10 mg IV glucagon, followed by infusion (2 mg/hour), 100 mL intralipid emulsion bolus, and by 1,200 mL/hour infusion. He also received three boluses of 8.4% sodium bicarbonate. He was then initiated on 50 mL 50% dextrose and insulin bolus (1 IU/kg), followed by infusion (0.5 IU/kg/hour). The patient was then intubated and veno-arterial ECMO was started after 4 hours. Methylene blue infusion at a rate of 1 mg/kg/hour was also initiated	The patient was decannulated after five days of ECMO initiation and extubated after 11 days. However, the patient sustained hypoxic brain injury, critical care polyneuropathy, and renal failure requiring dialysis. Four months after the overdose, he was still an inpatient, undergoing physical rehabilitation without the need for cardiac or respiratory support
Essink et al. [16]	Single pass albumin dialysis as rescue therapy for pediatric calcium channel blocker overdose	Case report	A 17-year-old female with an overdose of 550 mg of amlodipine	Fluid resuscitation, calcium infusion, and an insulin infusion at 0.1 U/kg/hour were initiated for hypotension, with improvement in her hemodynamics. She was transported to another center, and during the transport, doses of epinephrine and norepinephrine were initiated. Glucagon and sodium bicarbonate infusions were initiated on arrival. Insulin and calcium infusions were escalated. Veno-arterial ECMO was initiated after 9 hours. She also received ILE and methylene blue. SAPD was performed in series with ECMO using a CRRT machine, initiated on day three of the hospital stay, and the patient received SPAD for a total of 117 hours. She also received 23 hours of conventional CVVHD. Between days five and nine, glucagon, insulin, and calcium were stopped. Her inotropes were weaned off on day 11	The patient’s hemodynamics continued to worsen. Hypotension with mean arterial blood pressures between 42 and 55 mmHg persisted after ECMO initiation. She was stable hemodynamically for 12 hours after lipid emulsion. Methylene blue did not improve her hemodynamics. The patient developed severe fluid overload with skin tears following ongoing resuscitation. After 40 hours of SPAD, the patient improved clinically. She was decannulated from ECMO on day 11 and weaned off inotropes. She was extubated on day 12. She was shifted out of the ICU on day 16 and discharged on day 26 with the medical complication of a stroke with multiple small infarcts resulting in right-sided visual defects
Sampson and Bedy [17]	Lipid emulsion therapy given intraosseously in massive verapamil overdose	Case report	A 24-year-old female with a verapamil overdose (30 tablets of 240 mg extended-release verapamil)	Initially, the patient was given glucagon, high-dose insulin (100 U), and calcium gluconate. Then she was started on vasopressors (norepinephrine titrated up to 20 µg/minute) and 20% lipid emulsion therapy	Hemodynamic improvement was seen initially. The patient was admitted to the ICU and succumbed after 2 days
Ando et al. [18]	Re-elevation of serum amlodipine level after lipid emulsion therapy in an overdose case	Case report	A 73-year-old diabetic man with metformin (200 tablets 250 mg/tablet), glimepiride (78 tablets 3 mg/tablet), candesartan (29 tablets 8 mg/tablet), and amlodipine (84 tablets 5 mg/tablet) overdose	0.15 μg/kg/minute noradrenaline, 8.82 μg/kg/minute dopamine, 3.36 g of sodium carbohydrate solution (250 mL bag administered twice), 850 mg of calcium gluconate, 500 mL of 50% glucose solution, 3 mg/hour glucagon, and 8.3 mEq/hour KCl along with mechanical ventilation, followed by 100 mL of lipid emulsion formulation (20% intralipid) as bolus was administered. Hemodialysis and hemofiltration were also conducted for 24 hours	GCS improved to E3V2M4 in a few hours. After 30 hours, his hemodynamic status improved, and lactate levels, anion gap, and base excess stabilized. Mechanical ventilation was discontinued. He was discharged on the 10th post-hospitalization day
Ragot et al. [19]	Terlipressin in refractory shock induced by diltiazem poisoning	Case report	A 46-year-old woman with 8.4 g of extended-release diltiazem overdose	Insulin glucose and calcium were initiated, and norepinephrine was initiated to maintain MAP. Terlipressin was then started as the patient did not improve	Her hemodynamic state rapidly deteriorated. Acute oliguric renal failure started after 10 hours. Intermittent boluses of 0.1 mg of terlipressin lead to rapid improvement in hemodynamic status. Normal diuresis was recovered concurrently and quickly. Epinephrine and norepinephrine were stopped 11 and 46 hours after administration, respectively. On the 6th day, the patient was discharged
Connor-Schuler et al. [20]	The efficacy of albumin dialysis in the reversal of refractory vasoplegic shock due to amlodipine toxicity	Case series	Case 1: A 47-year-old male post-liver transplant for autoimmune hepatitis with amlodipine overdose (145mg). Case 2: A 39-year-old female with hypertension and a prior history of stroke with 600 mg amlodipine and 400 mg lisinopril overdose	Case 1: Norepinephrine, epinephrine, and vasopressin along with mechanical ventilation were initiated, followed by glucagon 10 mg, and then multiple 5 mg doses, four 250 mL doses of 20% fat emulsion, calcium gluconate infusion at 1.5 g/hour, hydrocortisone 100 mg, followed by 50 mg every six hours, and up-titration of an insulin infusion to 1 U/kg/hour with dextrose to maintain euglycemia, and, finally, albumin dialysis (molecular adsorbent recirculating system) with CRRT was initiated within 6 hours of admission. Case 2: She was given activated charcoal, glucagon, and IV fluids and was admitted to the ICU where she was given norepinephrine, epinephrine, vasopressin, and angiotensin II. She was given 16 g of calcium gluconate in divided doses, two 150 mg doses of methylene blue, three 50 mg doses of hydrocortisone, and a total of 770 mL of 20% fat emulsion divided over three doses. Insulin infusion was also started and rapidly up-titrated to 6 U/kg/hour with concurrent dextrose infusion to maintain euglycemia. She had metabolic acidosis that continued to worsen and was started on a bicarbonate drip and CRRT was done. She was then started on albumin dialysis. 20% intravenous ILE was started at 0.25 mL/kg/minute and continued for 3 hours	Case 1: Despite initial interventions, the patient remained hemodynamically unstable with a MAP of less than 65 mmHg. The patient’s hemodynamic status rapidly improved after albumin dialysis. He was extubated and transferred out of the ICU on day 3 of admission without any lasting organ system damage. Case 2: Despite interventions, the patient continued to be profoundly hypotensive with MAP in the 40s with worsening acidosis and rising lactate. After starting albumin dialysis, the patient’s hemodynamic status improved and she was discharged on the 12th day without any lasting organ damage
Mann [21]	Management of calcium channel overdose	Case report	A 48-year-old woman with polypharmacy overdose along with a diltiazem overdose	The patient was initially given 2 L of crystalloid solution, followed by atropine, calcium gluconate, and glucagon	The patient’s SBP increased to 90 mmHg and her heart rate was 60 bpm after initiating treatment, but she remained critically ill with persistent hypotension and an altered mental status. She was intubated for airway protection and had an arterial line inserted for continuous BP monitoring before her case was reviewed and she was transferred to the ICU for further management. Eight days after her initial presentation and against medical advice, she discharged herself from the ICU
Hubel and Bunin [22]	The sky’s the limit: high dose insulin for calcium channel blocker overdose	Case report	A 60-year-old male with hypertension. Amlodipine 300 mg with co-ingestion of Benzapril 300 mg	The patient was initially managed with glucagon, vasopressors, atropine, calcium chloride, and ILE. He was then initiated on insulin (90 IU/hour up to a maximum of 180 IU/hour) and dextrose infusion. He was intubated due to large volumes of dextrose needed to maintain euglycemia	The patient’s vital signs improved after the initiation of insulin dextrose, and vasopressor support was withdrawn after 96 hours. He was successfully extubated later
Ibrahim et al. [23]	Failure of hyperinsulinemic-euglycemia in calcium channel blocker overdose	Case report	A 56-year-old female with hypertension, depression, and a previous suicide attempt presented with a 105 mg amlodipine overdose	The patient was initially resuscitated with fluid, followed by norepinephrine and phenylephrine support. She was then started on only 0.5 U/kg short-acting insulin bolus, followed by a short-acting insulin drip starting at 0.5 U/kg/hour. She required multiple doses of glucose and potassium supplementation	Despite all measures, there was no marked increase in BP and she continued to require vasopressor support for the next 48 hours

Effects of Individual Treatment Modalities Used

Gastrointestinal decontamination: Four case reports utilized this treatment modality [4-7]. All reported cases of calcium channel blocker toxicity survived. In one case report, 70 g of activated charcoal followed by polyethylene glycol was used for gastrointestinal decontamination [4]. In the other two cases, gastric lavage with activated charcoal (60 g) was performed [5,6]. None of the cases reported cardiac arrests or aspiration after gastric lavage. In one case report, gastrointestinal decontamination was done using magnesium sulfate [7].

High-dose insulin euglycemic therapy (HIET): High-dose insulin intravenous bolus is given at a dose of 1 unit/kg/hour followed by 0.5 to 2 units/kg/hour infusion. This modality was utilized by all 20 studies including one original article. Three case reports showed negative results and the patients succumbed [8-10]. In the original article, 66 calcium channel blocker overdose patients were reported. All these patients received high-dose insulin therapy, out of which 36.3% of patients were reported to develop hypokalemia and 25.7% developed hypoglycemia [11]. The majority of the studies reported improvement in hemodynamic parameters in the form of blood pressure, heart rate, mean arterial pressure, and urine output with HIET [4-7,9,11-20].

Injection calcium gluconate or calcium chloride: In almost all case reports, the usage of injection calcium gluconate or calcium chloride was employed. However, inconsistent effects were reported. Improvements in blood pressure and heart rate were reported [4-7,12-16,19-22]. Two patients expired despite the use of calcium gluconate [8,9]. The reported dosage of calcium gluconate used was in the range of 1 to 5 g.

Vasopressors: The most common vasopressor used in calcium channel blocker-induced cardiogenic shock was norepinephrine [4-10,12-20,22]. In one study, phenylephrine was utilized, and terlipressin was used in another [19]. Other vasopressors used were dopamine and vasopressin. Isoprenaline was used in one case report to improve the heart rate [7]. In another case report, aliquots of adrenaline (100 µg) were used, followed by epinephrine infusion (0.1 µg/kg/minute) and dobutamine (3.13 µg/kg/minute). The patient showed improvement in hemodynamic parameters [15]. The use of both noradrenaline and dopamine showed improvement in the survival of the patients, as well as hemodynamic improvements such as a rise in blood pressure and heart rate.

Methylene blue: The use of methylene blue at a dose of 1 mg/kg/hour was reported in three cases to improve blood pressure. All three patients survived and reported improvements in hemodynamic parameters [13,15,16].

Glucagon: Glucagon was used in the dose of 9-10 mg intravenous bolus followed by 5-7 mg/hour infusion. The use of the same was reported in 16 cases. All except two cases reported improvement in both the blood pressure and heart rate, while in two cases, hyperglycemia and vomiting were reported post-glucagon therapy [4-10,12-16,20-22].

Intravenous lipid emulsion (ILE) therapy:20% ILE therapy was utilized at a dose of 0.25 mL/kg/minute for three hours in one case report [20], while a dose of 100 mL bolus followed by 1,200 mL/hour was considered in another [15]. ILE was used as a treatment option in six case reports and one case series [8,9,12,13,17,18,20]. All except one study showed improvement in blood pressure and heart rate [8].

Single-pass albumin dialysis: It was utilized in two case studies as a treatment measure for calcium channel blocker toxicity [16,20]. Both case studies showed benefits to the patient outcome with the use of albumin dialysis.

Extracorporeal membrane oxygenation (ECMO): The use of ECMO was found to be associated with an improvement in survival in patients with calcium channel blocker toxicity. Arterial-venous ECMO was used in five case reports [6,8,13,15,16]. Out of these cases, one patient did not survive [8]. The use of ECMO was associated with increased chances of survival and improvement in the left ventricular function and blood pressure.

Effects of Interventions on the Outcome of the Patients

Effect on mortality: HIET was associated with improved survival reports. In most case studies, it was given after the injection of calcium gluconate and glucagon therapy. It was utilized in all 20 articles included in the systematic review, including the cohort study. Overall, 20% ILE therapy was also associated with better survival and was used in eight case studies after high-dose insulin therapy. Vasopressors such as noradrenaline and dopamine showed improvement in mortality rate but the results were inconsistent. ECMO was used in patients with refractory shock as the last therapeutic resort. It was also associated with better survival in these patients [6,13,15,16]. Two patients survived after receiving albumin dialysis [16,20].

Effects on hemodynamic parameters: Positive effects were reported with glucagon, calcium gluconate, high-dose insulin therapy, as well as lipid emulsion. The case studies showed improvement in blood pressure, heart rate, mean arterial blood pressure, and urine output. The case studies also showed the positive effects of methylene blue on blood pressure [13,15,16]. This review also highlights the role of vasopressors in the improvement of blood pressure for patients with calcium channel blocker overdose.

Improvement in functional status: One case report documented that the patient was discharged without any lasting organ system damage [20]. This case report described two cases of calcium channel blocker poisoning. The patients underwent albumin dialysis apart from the usual treatment given. In another case report, the patient was discharged with an intact mental status and returned to functional independence on day 56. Veno-arterial ECMO was used for the patient for the management of a massive amlodipine overdose [13]. Koliastasis et al. reported a case with refractory shock from an amlodipine overdose overcome with hyperinsulinemia. The patient was discharged on day 14 and on follow-up at one month showed no cardiovascular comorbidity [5]. Cole et al. conducted a study among 41 patients who experienced cardiac arrest at some point during the treatment [11]. Another case report highlighting the efficacy of albumin dialysis in the reversal of refractory vasoplegic shock due to amlodipine toxicity reported that the patient was discharged on day 12 without any lasting organ dysfunction [20].

Study limitations

The major limitation of our study was the heterogeneity of the available data. As most of the studies included in our review were case reports, the risk of bias could not be determined. The lack of any randomized control trials for the individual or all treatment modalities makes it difficult to analyze the exact effects of the same in the management of calcium channel blocker poisoning.

## Conclusions

This systematic review focuses on the array of treatment modalities available for calcium channel blocker toxicity and their effects on mortality and other desired functional outcomes. Multiple treatment modalities are available such as calcium, high-dose insulin, lipid emulsion, vasopressors, ECMO, and albumin dialysis. As controlled trials are lacking, the evidence supporting superiority is inconsistent. Thus, randomized controlled trials supporting their role should be conducted. However, due to ethical concerns, in most toxicological emergencies, conducting randomized controlled trials may not be feasible.
