# Correction: Treatment Modalities in Calcium Channel Blocker Overdose: A Systematic Review

**DOI:** 10.7759/cureus.c269

**Published:** 2025-08-26

**Authors:** Himanshi Baid, Nidhi Kaeley, Shiana Singh, Prakash Mahala, Hannah Chawang, Soumya Subhra Datta, Harsimran Manchanda, Takshak Shankar

**Affiliations:** 1 Department of Emergency Medicine, All India Institute of Medical Sciences, Rishikesh, Rishikesh, IND

This article has been corrected to fix the following typos present in the PRISMA diagram:

- "Reports excluded: animal studies (n=**51**)" has been corrected to "Reports excluded: animal studies (n=**50**)"
- "Reports excluded: mixed unknown substance poisoning (n=**384**)" has been corrected to "Reports excluded: mixed unknown substance poisoning (n=**364**)"

